# The Management of Postoperative Cognitive Dysfunction in Cirrhotic Patients: An Overview of the Literature

**DOI:** 10.3390/medicina59030465

**Published:** 2023-02-26

**Authors:** Daiana-Georgiana Ingustu, Bogdan Pavel, Silvia-Ioana Paltineanu, Diana-Irene Mihai, Mihail Cotorogea-Simion, Cristina Martac, Madalina-Marieta Florescu, Cristian Cobilinschi, Sebastian Isac, Gabriela Droc

**Affiliations:** 1 Department of Anesthesiology and Intensive Care I, Fundeni Clinical Institute, 022328 Bucharest, Romania; 2 Department of Physiology, Faculty of Medicine, Carol Davila University of Medicine and Pharmacy, 020021 Bucharest, Romania; 3 Department of Gastroenterology, “Saint Mary” Clinical Hospital, 011172 Bucharest, Romania; 4 Department of Anesthesiology and Intensive Care, Clinical Emergency Hospital of Bucharest, 14461 Bucharest, Romania; 5 Department of Anesthesiology and Intensive Care II, Carol Davila University of Medicine and Pharmacy, 050474 Bucharest, Romania; 6 Department of Anesthesiology and Intensive Care I, Carol Davila University of Medicine and Pharmacy, 020021 Bucharest, Romania

**Keywords:** postoperative cognitive dysfunction, cirrhosis, hepatic encephalopathy, general anesthesia, depth of anesthesia, perioperative management in liver cirrhosis, perioperative virtual reality

## Abstract

*Background and objectives*: Postoperative cognitive dysfunction (POCD) represents a decreased cognitive performance in patients undergoing general anesthesia for major surgery. Since liver cirrhosis is associated with high mortality and morbidity rates, cirrhotic patients also assemble many risk factors for POCD. Therefore, preserving cognition after major surgery is a priority, especially in this group of patients. The purpose of this review is to summarize the current knowledge regarding the effectiveness of perioperative therapeutic strategies in terms of cognitive dysfunction reduction. *Data Collection*: Using medical search engines such as PubMed, Google Scholar, and Cochrane library, we analyzed articles on topics such as: POCD, perioperative management in patients with cirrhosis, hepatic encephalopathy, general anesthesia in patients with liver cirrhosis, depth of anesthesia, virtual reality in perioperative settings. We included 115 relevant original articles, reviews and meta-analyses, and other article types such as case reports, guidelines, editorials, and medical books. *Results*: According to the reviewed literature, the predictive capacity of the common clinical tools used to quantify cognitive dysfunction in cirrhotic settings is reduced in perioperative settings; however, novel neuropsychological tools could manage to better identify the subclinical forms of perioperative cognitive impairments in cirrhotic patients. Moreover, patients with preoperative hepatic encephalopathy could benefit from specific preventive strategies aimed to reduce the risk of further neurocognitive deterioration. Intraoperatively, the adequate monitoring of the anesthesia depth, appropriate anesthetics use, and an opioid-sparing technique have shown favorable results in terms of POCD. Early recovery after surgery (ERAS) protocols should be implemented in the postoperative setting. Other pharmacological strategies provided conflicting results in reducing POCD in cirrhotic patients. *Conclusions*: The perioperative management of the cognitive function of cirrhotic patients is challenging for anesthesia providers, with specific and targeted therapies for POCD still sparse. Therefore, the implementation of preventive strategies appears to remain the optimal attitude. Further research is needed for a better understanding of POCD, especially in cirrhotic patients.

## 1. Introduction

Nowadays, major abdominal surgery represents one of the most common occasions for exposure to general anesthesia. Bowel, liver, pancreatic surgery, or various oncologic surgical procedures for pelvic tumors exposes the patient usually hours to surgical stress and anesthesia. Due to the increased life expectancy, elderly patients with chronic medical conditions, such as liver cirrhosis, are prone to developing various postoperative complications, including postoperative cognitive dysfunction (POCD) [1,2,3].

POCD consists of decreased cognitive performance in patients undergoing general anesthesia during major surgery. Its pathophysiological mechanisms are not well known, but it is believed that approximately one-quarter of older individuals undergoing major surgery will suffer from cognitive decline, of which 50% will have permanent dysfunction [1].

**Figure 1:**
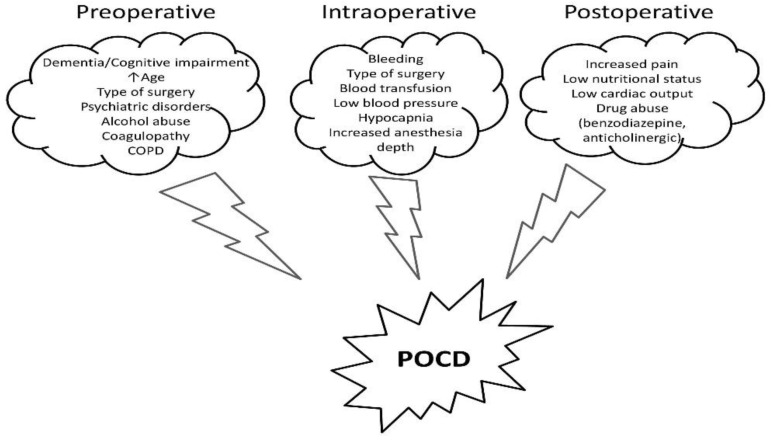
Risk factors and intervention strategies for POCD. POCD—postoperative cognitive dysfunction, COPD—chronic obstructive pulmonary disease.

The risk factors for POCD may be grouped into preoperative, intraoperative, and postoperative (Figure 1). Dementia, age, level of education, type of surgery, chronic obstructive pulmonary disease (COPD), psychiatric or degenerative disorders, alcohol, and illicit drug use are the most common preoperative risk factors [2,3] Intraoperative risk factors include bleeding (greater than 1000 mL), hypotension, hypocapnia, and depth of anesthesia [2,3]. The main postoperative risk factors are severe pain, benzodiazepines, and anticholinergic drugs, inadequate nutritional status, and low cardiac output [2,3].

POCD is usually transient and detectable with appropriate testing through comparison with preoperative cognition. It affects memory, learning abilities, perception, attention, executive functions, and verbal abilities and should be differentiated from postoperative delirium (POD) [4,5]. Usually, cirrhotic patients assemble many of the above-mentioned risk factors for developing POCD: preexisting hepatic encephalopathy (HE), alcohol and/or illicit drug use, poor nutritional status, cirrhotic coagulopathy, incomplete clearance of the endogenous benzodiazepines, hypotension, and modified drug pharmacokinetics. Thus, preserving cognition in cirrhotic patients after major surgery should be prioritized. Various pathophysiological pathways could lead to POCD-related impairments in cirrhotic patients: hyperammonemia, endotoxemia, chronic inflammation, and brain edema [6,7,8,9].

Hepatic encephalopathy (HE) is one of the various complications of end-stage liver disease and it represents a major clinical problem in assessing liver cirrhosis. Symptoms may vary from mild impairment in mental state, only diagnosed through psychometric tests, to coma [10,11]. Due to challenges in diagnosing minimal HE (mHE), its prevalence and incidence are hard to predict, but it is believed to affect approximately 40% of cirrhotic patients worldwide [12]. The first classification of HE was made in 1998 by the World Organization of Gastroenterology and modified by the European Association for the Study of the Liver-American Association for the Study of Liver Diseases (EASL-AASLD) consensus in 2014. It was categorized according to etiology, severity, time course, and occurrence (precipitated vs. spontaneous) [11]. For clinical purposes, West Haven criteria are used to classify HE into four stages, but they are often used subjectively and fail to accurately identify minimal hepatic encephalopathy (mHE). Thus, further efforts should be made to identify novel predictive biomarkers in various subclinical cirrhosis-associated conditions that could lead to cognitive impairment [8,9,10,11,12,13,14].

**Figure 2:**
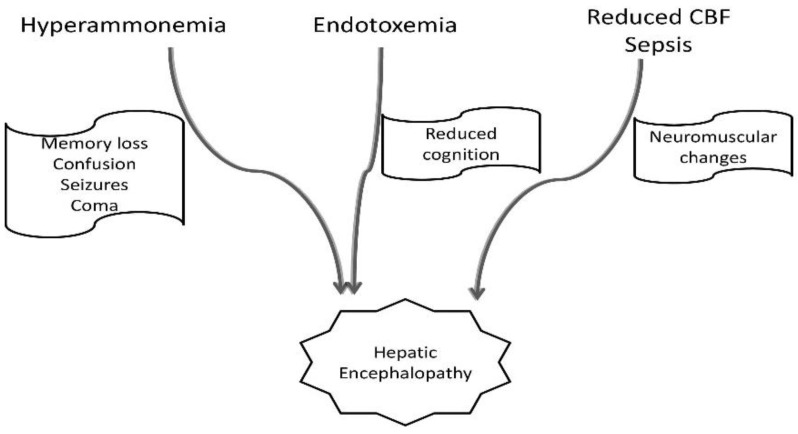
The mechanisms of hepatic encephalopathy in cirrhotic patients.

HE is proven to be the result of excessive amounts of ammonia produced by the liver [6,8,9,11]. Hyperammonemia leads to neurocognitive dysfunction consisting of memory loss, shortened attention span, confusion, agitation, changes in personality, seizures, and ultimately coma, regardless of anesthesia exposure [8,12]. Other neurological manifestations consist of neuromuscular changes (asterixis, paratonic rigidity, hyperreflexia, and tremor), believed to be the result of cerebral blood flow alterations, accumulation of brain metabolites, and release of inflammatory mediators [9]. Another consequence of liver failure is portosystemic shunting, leading to endotoxemia and consequently to reduced cognition [10,15]. Inflammation also plays a major role in the pathophysiology of HE [6,7,9,10,11]. In chronic liver dysfunction patients, urea synthesis is impaired, so astrocytes from the brain act as an alternate pathway for ammonia detoxification, resulting in the accumulation of glutamine within astrocytes, leading to swelling [9]. Mild diffuse brain edema was found in patients with the mHE using magnetic resonance imaging [16,17]. Diffuse white matter anomalies have been detected with magnetization transfer ratio measurements and fast-Flair sequences. Those abnormalities were found in the absence of clinical signs of HE and were reversible after liver transplantation surgery [16]. Animal and human models were used to prove that ammonia causes HE only in the presence of systemic inflammatory response syndrome (SIRS) [9]. Sepsis may trigger HE, due to altered nitrogen metabolism and the presence of an increased inflammatory response [7]. The possible mechanisms for HE are revealed in Figure 2.

Liver transplant is the only cure for liver cirrhosis, but preoperative HE may have negative effects on postoperative outcomes [18,19]. It was initially believed that HE symptoms are temporary, but new research shows that many neuropsychiatric and neuromuscular manifestations tend to have a long-term or permanent effect on the quality of life in these patients [18]. Neurological complications may affect approximately 75% of patients in the first month after transplantation, suggesting a link between preoperative status and the postoperative cognitive dysfunction seen in many older individuals after major surgery [19]. Neurologic symptoms were noticed even 1 year after the transplant, due to a variety of factors such as sepsis, the persistence of portosystemic shunts, and immunosuppressant-associated toxicity [18].

According to the EASL (European Association for the Study of the Liver) guideline, HE should be prevented with standard therapy first, by prescribing non-systemic antibiotics (e.g., rifaximin) and non-absorbable disaccharides (e.g., lactulose) [20]. Airway patency should also be preserved to prevent aspiration pneumonia. If possible, HE should be corrected prior to general anesthesia, as it may cause POCD. For better prophylaxis of POCD in cirrhotic patients, various risk factors for HE should also be considered and treated, as they may worsen the postoperative neurocognitive status of the patient and decrease survival [20].

The aims of this review are to summarize and evaluate the impact of perioperative cognitive impairment in cirrhotic patients and to highlight new therapeutic strategies to reduce its deleterious effects.

## 2. Methodology

**Figure 3:**
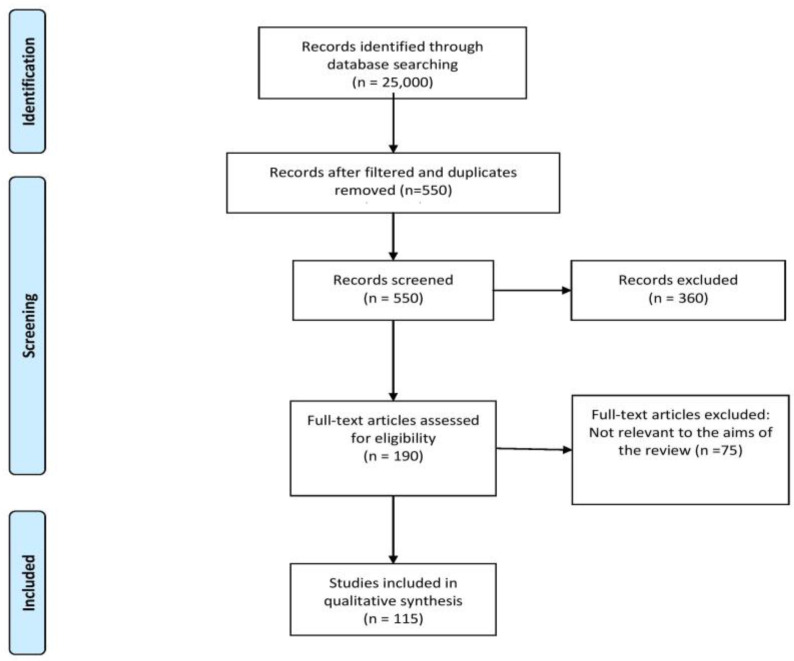
PRISMA- 2020 flow diagram for selection of studies.

We reviewed articles using various medical search engines including PubMed, Google Scholar, and Cochrane library, published from 2000 up to December 2022. We considered the following keywords: POCD, perioperative management in patients with cirrhosis, HE, general anesthesia in patients with liver cirrhosis, depth of anesthesia, and virtual reality in perioperative settings. The search engines delivered a total of 25,000 articles. After filtering and duplicates removal we identified 550 articles. We checked for eligibility and further excluded 360 articles. From the remaining 190 full-text articles, 115 articles were included, based on novelty and relevance (Figure 3).

## 3. Perioperative Cognitive Dysfunction Management in Cirrhotic Patients

With the increasing prevalence of chronic liver disease, up to 10% of cirrhotic patients may have non-transplant surgery within the last two years of their lives [21]. Depending on the degree of liver disease, perioperative mortality is generally 2–10 times higher in individuals with cirrhosis than in those without [22,23]. In cases of decompensated cirrhosis, general anesthesia and surgery might result in severe morbidity and significant perioperative mortality. The most significant clinical tools in identifying cirrhotic patients at risk for surgery and anesthesia, such as the MELD score, do not include, however, the presence of perioperative cognitive dysfunction, which can lead to prolonged ICU stay and, additionally, increase mortality [24,25]. Hemodynamic abnormalities, which are more obvious than all others, could occur as an inappropriate response to surgical stress due to the minimal hepatic reserve and the systemic disturbances brought on by liver dysfunction [24]. Furthermore, inappropriate blood flow to the brain along with disturbed liver metabolism could aggravate a subclinical cognitive dysfunction. Thus, supplementary perioperative complex cognitive assessments should be implemented, along with therapeutic strategies to mitigate this potentially dangerous complication.

**Figure 4:**
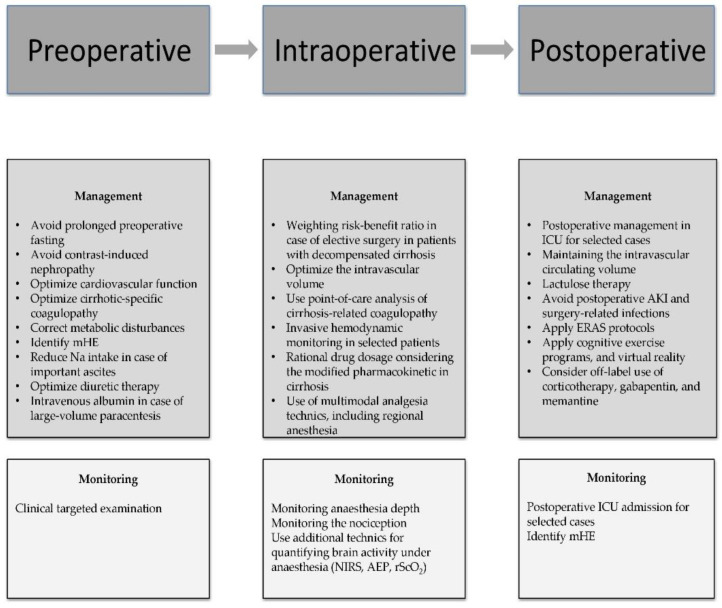
The main perioperative strategies for reducing POCD in cirrhotic patients. Abbreviations: mHE—minimal hepatic encephalopathy, NIRS—near-infrared spectroscopy, AEP—auditory evoked potentials, rScO_2_—the regional cerebral oxygen saturation, ICU—Intensive care unit, AKI—acute kidney injury, ERAS—early recovery after surgery.

The clinician’s efforts should focus on the three main critical care stages: preoperative, intraoperative, and postoperative period, in order to develop new innovative strategies to reduce cognitive impairment in patients with end-stage liver disease. The most important perioperative strategies are summarized in Figure 4.

### 3.1. Preoperative Management of Cognitive Dysfunction in Cirrhotic Patients

#### 3.1.1. Pathophysiologic Mechanisms of Cognitive Impairment in Cirrhotic Patients

According to the literature, age, ASA classification, and the degree of liver disease as mirrored by the MELD score are the three most significant predictors of mortality [25]. Nevertheless, emergency surgery has been linked to greater rates of morbidity and death rates than elective surgery in cirrhotic patients [24,26]. Even if no direct causality was observed between the degree of surgical emergency and cognitive decline, various perioperative complications such as hypotension, renal failure, and coagulation abnormalities could precipitate POCD in cirrhotic patients exposed to emergency surgery [27]. The most frequent indications for emergency surgery in cirrhotic patients are gallstones, abdominal wall hernia, small bowel, appendix, colorectal, or gastric surgery [24,28,29]. Thus, the clinician’s ability to predict and treat cirrhosis-associated comorbidities, regardless of the urgency grade, poses a central role in reducing the POCD rate.

A study gathering data from 30,927 patients undergoing colorectal resection surgery demonstrates higher rates of death and major perioperative complications in patients with cirrhosis compared to patients without chronic liver disease [30].

The examination of the patient with liver disease should start with a rigorous history and clinical examination, with close attention to any remaining factors that precipitate cirrhosis, such as prior blood transfusions, jaundice, hepatosplenomegaly, or ascites [31].

Some of the major abnormalities that could precipitate cognitive impairment and should be reviewed and corrected in cirrhotic patients are the history of bleeding, coagulopathy, mHE, metabolic disturbances, hepatorenal and hepatopulmonary syndrome (HPS), and hepatic cardiomyopathy [32].

The link between cerebral hemodynamics and cognitive function in the cirrhotic patient was already established, so further attention should be focused on the cardiovascular function prior to surgery [33,34]. Patients with chronic liver disease have hyperdynamic circulation with an increased risk to develop cirrhotic cardiomyopathy [22,35]. A review from 2010 illustrates that hyperdynamic circulation is associated with impaired perfusion of many organs, including the brain, in cirrhotic patients [35].

According to a recent experimental study, brain oxygenation could be significantly impaired in rodents with mHE [34,36]. Moreover, hypoxemia in cirrhotic patients is a common issue. Pleural and peritoneal effusions or HPS are the most common causes of hypoxemia and secondary brain hypoxia [22]. HPS implies a high risk for patients undergoing non-liver transplantation surgery and it should be excluded by performing contrast echocardiography [22,37].

The association of hyperammonemia and uremia is detrimental to brain function in cirrhotic patients. Preoperative negative fluid balance, prolonged preoperative fasting, and contrast-induced nephropathy are the most important precipitating factors for kidney injury [22].

The presence of ascites is relevant to abdominal surgery because of the large volume of fluid that is eliminated by paracentesis. The causality between large-volume paracentesis and HE is well established, therefore, prior to surgery, a patient with significant ascites might benefit from reducing the sodium intake, diuretic therapy, and intravenous albumin [38,39]. Regular coagulation tests, thromboelastography, and platelet count should be performed before abdominal surgery in patients with chronic liver disease because intraoperative bleeding and blood transfusions are independent risk factors for postoperative cognitive impairment [22,40].

Patients with cirrhosis are prone to developing cognitive disorders in the perioperative period, due to various direct mechanisms, regardless of the above-mentioned associated organ dysfunctions [41]. POCD is considered to be a complication that occurs, usually, in the first weeks following surgery (10–54%) [42]. The patient-related risk factors for developing POCD are previous mental health or cognitive dysfunction (including HE), age, and level of education [1]. A recent systematic review pointed out the importance of cognitive reserve in the postoperative outcome of cognitive disorders. Their results showed that subjects with a higher education level have a lower risk of developing POCD [34].

A prospective study showed that, in patients with cirrhosis, infection was an independent predictive factor for cognitive dysfunction. Cognitive alterations, clinical or subclinical, were identified in 42% of the subjects with cirrhosis but no infection, 79% of cirrhotic patients with infection but no SIRS, and 90% of the patients with sepsis [43].

HE may be present preoperatively, in a clinical or subclinical form. Thus, recognizing and treating HE is an important step in preoperative management. Untreated, HE can worsen the postoperative outcome by causing respiratory complications (aspiration pneumonia), and immobility, increasing the need for unnecessary investigations and making the patient uncooperative to further medical procedures [44]. HE has a more fluctuating course compared to neurodegenerative disorders such as cerebrovascular diseases or dementia [45].

HE symptoms have an acute onset, with disinhibition and changes In personality. The risk of developing HE is high in patients with previous cognitive disorders [45]. One study from 2010, which evaluated 226 cirrhotic patients with and without HE, showed that, despite adequate therapy after the first episode of encephalopathy, cognitive function remains affected, especially learning capacity, working memory, and executive functions [46]. Another article from 2011, analyzing 106 patients with cirrhosis, highlights the partial reversibility of neurological impairment after HE episodes [47].

#### 3.1.2. Preoperative Assessment of Cognitive Dysfunction in Cirrhotic Patients

Two of the most known scoring systems for predicting the outcome of cirrhotic patients undergoing surgery are the Model for End-Stage Liver Disease (MELD) and Child-Turcotte-Pugh (CTP) [27]. Both scores focus more on the general perioperative mortality in cirrhotic patients and to a lesser extent on the cognitive decline after surgery.

The CTP score lacks reproducibility because it relies on the observer’s perspective of the HE and ascites [27]. There are several studies that link the increasing mortality rate to Child classes A, B, and C. [27]. Telem et al. noticed that patients with cirrhosis undergoing abdominal surgery had lower mortality rates, 2% for CTP class A, and 12% for both CTP class B and C [48].

The MELD score takes into consideration the patient’s serum creatinine, bilirubin, and INR. These are objective laboratory parameters therefore the MELD score is more reproducible than the CTP score in terms of mortality but does not include any parameters for the evaluation of cognition [27]. Perkins et al. discovered that patients with a MELD score over eight have higher postoperative morbidity [49]. A study analyzing 140 surgical procedures in patients with cirrhosis showed that mortality is proportional with each point in the MELD score (1% increase until 20 points and 2% thereafter) [50].

There are several studies that compared the sensitivity of the MELD and CTP scores in relation to perioperative morbidity and mortality in patients with cirrhosis. Befeleret al. observed that a cut-off value for a MELD score of 14 was a more reliable predictor of poor postoperative outcome than CTP class C when considering abdominal surgery in cirrhotic patients [51]. Delis et al. showed that the rate of postoperative complications was higher in cases with a MELD score over 13, while for the CTP score, there was no correlation with postoperative morbidity [52].

Conversely, for the evaluation of cognition prior to surgery in cirrhotic patients, no clinical tools reached a convenient sensitivity. Various neuropsychological tests could assess, however, the presence of HE and cognitive dysfunction. Pantiga et al. performed a study that included 150 subjects divided into five groups: Child A, B, and C, patients with a history of liver transplantation, and the control group.

They used five scores to assess memory loss, attention deficit, ability to reason, thinking, and disorientation: Digit Span Forward (DF), Digit Span Backward (DB), Raven’s Progressive Matrices Test (RT), and Trail Making Test A and B (TMT-A, B). Differences in cognition were recorded between the five groups, revealing that cognitive dysfunction increased with the progression of liver disease. In patients with a history of liver transplantation, the degree of cognitive impairment is slightly higher than in the control group but lower than in patients with Child A, B, or C cirrhosis. Their study proved that neuropsychological scores were well-suited for assessing HE [53].

Campagna et al. examined if the Animal Naming Test (ANT) might be used for HE screening. They divided the patients into three groups: a group of healthy subjects for the standardization of ANT, a control group constituted of inflammatory bowel disease patients, and the final group made up of cirrhotic patients. The results pointed out that age and the level of education influenced the ANT score in healthy individuals. Conversely, in cirrhotic patients, the ANT score was proved to be inversely proportional to the level of serum ammonia and the MELD score. Patients with previous episodes of HE grade 2 or above scored lower than those who did not have a history of HE. Patients with minimal HE scored higher than subjects with HE grade 1. They concluded that the ANT test was a useful, low-cost, fast tool for investigating HE [54].

### 3.2. Intraoperative Management of Cognitive Dysfunction in the Cirrhotic Patient

In addition to the significant risk of morbidity and mortality after anesthesia and surgery in cirrhosis, any preexistent cognitive deficit could influence postoperative care. The direct effect of anesthesia on the brain is still debatable [55,56]. Furthermore, specific novel biomarkers in cirrhosis could identify new predictive algorithms in patients at risk [14].

Elective interventions should be performed only in cases with compensated chronic liver disease [14,57]. Postponing elective surgery in patients with decompensated liver cirrhosis should be considered by weighting the risk-benefit ratio. Nevertheless, according to a study published by Friedman, there are several clinical conditions that contraindicate any elective surgery: fulminant hepatic failure, acute viral or alcoholic hepatitis, Child’s class C cirrhosis, severe coagulopathy, hypoxemia, cardiomyopathy, or acute renal failure [58]. Moreover, particular attention must be given to the neurological status of the patient and special measures taken in the perioperative period in order to minimize POCD, even if no consistent recommendation exists with regard to postponing an elective surgery based on the presence of HE or any other cognitive decline alone [59,60]. Intraoperative management poses a few challenges, such as optimization of the intravascular volume in the presence of ascites and peripheral edema. Goal-directed fluid therapy and avoiding overloading appear to be beneficial [1]. Blood products are routinely used in these cases and employment of point-of-care analysis of coagulopathy, such as ROTEM or TEG, proves to be an optimal approach [61]. Moreover, most cirrhotic patients are under treatment with β-blockers. Thus, their cardiac response to stress may be reduced. Therefore, it is advisable that all cirrhotic patients benefit from standard monitoring and that, in selected cases, especially in the setting of major surgery, invasive monitoring be used with the aim of better managing the frequently occurring intraoperative hypotension, which is described as a risk factor of POCD [60,61].

Evaluating baseline cognitive function may prove useful in identifying patients at risk of developing POCD, as preoperative cognitive impairment is correlated with an increased probability of POCD [1]. Anesthesia and surgery-related risk factors for POCD refer to adequate analgesia or hypnosis, reduced blood flow to the brain, massive bleeding with an increased volume shift, and prolonged duration of the surgery [1]. Therefore, specific anesthetic strategies may be implemented in an attempt to minimize POCD. Intraoperative management becomes crucial in the health practitioners’ pursuit of maximizing postoperative brain health.

Liver dysfunction associated with cirrhosis results in the alteration of all pharmacokinetic phases: absorption, distribution, and elimination [62]. Impaired liver function translates to increased portosystemic pressure, which increases the bioavailability of the drugs as a result of reduced first-pass metabolism. Diminished protein synthesis function, with lower serum albumin concentrations, as well as ascites formation and volume overload, explain the changes in the distribution phase in these patients [62]. Therefore, cirrhosis implies a higher volume of distribution for both highly protein-bound drugs and water-soluble drugs. The elimination phase is also subject to changes in liver disease, with different degrees of impairment of the various metabolizing enzymes, the cytochrome P450 system being more severely affected [62]. Consequently, multimodal analgesia techniques should be used in order to reduce drug dosage and the incidence of POCD [63]. Of particular interest is the use of regional anesthesia in cirrhotic patients. Regarding epidural analgesia, coagulation-related complications may prevent its use in cirrhotic patients. However, in cases with normal coagulation, especially in those cases where ROTEM or TEG analysis were performed, regional anesthesia may be safe [61]. Better pain management, reduced complication rates, and enhanced recovery are among the added benefits of epidural anesthesia [64].

A systematic review analyzing seven studies and 1031 patients described a short-term benefit of regional anesthesia on POCD, in the first 3 days after surgery. However, no long-term benefits were mentioned [65]. Moreover, a randomized study by Rasmussen et al. found that regional anesthesia may decrease POCD incidence in the early postoperative period, but no difference was reported at 3 months [66]. Another study showed similar POCD incidence, regardless of epidural anesthesia. However, the authors identified better linguistic function and memory in patients with combined general and epidural anesthesia. The improved pain control might be of benefit to this group, preventing cognitive dysfunction [67]. On the contrary, Silbert et al. found no consistent benefits of regional anesthesia regarding POCD and do not recommend the use of a regional anesthesia technique instead of or in combination with general anesthesia in order to minimize POCD incidence [4].

Various common anesthetic drugs such as volatile anesthetics, IV hypnotics, opioids, and neuromuscular blocking agents have controversial impacts on POCD incidence in cirrhotic patients.

Inhalational hypnotics are widely used for general anesthesia. These could reduce hepatic blood flow, with consequent additional hepatic injury. Marked hepatic injury incidence related to the use of newer inhalational anesthetic agents has been reported, however, to be very low [62]. Since halothane is, nowadays, unavailable in almost all countries, new agents such as sevoflurane, and desflurane, are viewed as safe options in cirrhotic patients, undergoing minimal hepatic metabolism. Desflurane was reported to be the best preserver of liver blood flow [59,60,62]. It is widely accepted that, even though none of the newer volatile agents has been particularly associated with an increased risk of POCD, there is an evident age-dependent change in sensitivity to these inhalational agents. The MAC of an agent is known to be subject to change, declining by 6% every ten years after the age of 30. Thus, the CODA trial showed that a reduction of 39% in the age-adjusted end-tidal MAC was correlated with a 35% decrease in POCD [68]. The cognitive dysfunction after exposure to volatile agents may be caused by the deposition of β-amyloid, changes in neurotransmission, or an increase in the pro-inflammatory cytokines [69]. Sevoflurane and desflurane also proved to be more advantageous than other inhalational agents as part of the prevention strategies of POCD [1].

Propofol is a widely used IV hypnotic, with a protein binding proportion of 97–99%. It is used for both induction and maintenance of anesthesia and does not show any major changes in pharmacokinetics other than a larger volume of distribution in cirrhotic patients. Due to its short half-life, it remains the drug of choice even in decompensated cirrhosis [59,62]. However, a dose reduction might be required as a consequence of cirrhotic patients’ exacerbated cardiovascular side effects [70]. Propofol is generally accepted as a safe drug for the prevention of POCD [1]. The neuroprotective effects are explained by its antioxidant qualities, as well as its role in glutamate neurotransmission modulation [70]. Thus, total intravenous anesthesia (TIVA) may be a safe option for patients with altered liver function [71]. Regarding POCD, a meta-analysis involving 753 patients comparing propofol anesthesia with volatile anesthesia revealed that propofol is associated with a lower risk of POCD [72]. Moreover, a Cochrane review from 2018 found that propofol-based TIVA may reduce the risk of POCD when compared to inhalational maintenance of anesthesia [73]. According to Qiao et al., propofol reduces astrocytic IL-6 and TNF-α production, promoting synaptic inhibition, while volatile anesthesia has the opposite effect. This mechanism could be involved in the increased risk of POCD [74]. Propofol proved to be superior to both dexmedetomidine and midazolam in reducing short-term POCD incidence [75]. These findings may be even more evident in cirrhotic patients, given the high protein binding and predominant hepatic elimination of midazolam [62].

Dexmedetomidine, an α2-adrenergic agonist with predominant hepatic elimination and potential neuroprotective benefits in animal models, has been reported to decrease the risk of POCD in both cardiac and non-cardiac surgeries [76,77]. Dexmedetomidine is, however, used often as a sedative in ICU settings and to a lesser extent perioperatively. Another study by Deiner et al. showed that a significant reduction in delirium and POCD was associated with postoperative administration and not intraoperative infusion [78]. In liver failure patients, the dose must be adjusted. Dexmedetomidine used during anesthesia in cirrhotic patients induced an attenuated stress response, a decrease in inflammation levels, and reduced opioid consumption, as well as improved liver function [79].

Ketamine, an N-methyl-D-aspartate antagonist, may prove useful in the prevention of POCD [80]. In contrast, a randomized clinical trial exhibited evidence that the use of a single subanesthetic dose of Ketamine has no benefit in averting POCD or reducing postoperative pain. Moreover, it may be detrimental as it may increase the incidence of hallucinations in the postoperative period [81]. Results of single bolus administration are inconsistent; therefore, infusion regimens may be a potential subject for studies in an attempt to evaluate ketamine’s neuroprotective effects [65].

Opioids are widely used in the intraoperative management of cirrhotic patients. Pharmacokinetic particularities in cirrhosis consist of prolonged half-life and altered clearance. Fentanyl is reported to be the opioid of choice in this setting [59]. The dosing regimen is not altered with the exception of severe hepatic dysfunction, as its metabolism occurs almost entirely in the liver [64]. Nevertheless, large or repeated doses may lead to accumulation [60]. Remifentanil is a valuable option because it is metabolized by tissue esterases, which are preserved even in the context of hepatic dysfunction. There is widespread agreement that morphine should be avoided in decompensated cirrhotic patients as it may act as a trigger for HE occurrence [60]. Moreover, opioid-free anesthesia is reported to improve patients’ outcomes through better management of postoperative pain as opioid use is linked to secondary hyperalgesia [82].

Neuromuscular blockers (NMBs) could have their effects prolonged in liver cirrhosis patients, due to the particular pharmacokinetics associated with hepatic dysfunction. Depolarizing NMBs, such as succinylcholine, cause prolonged paralysis due to the reduced pseudocholinesterase activity associated with liver impairment [62]. However, this has a minor impact on everyday practice and dose changes are not recommended [60,62]. Non-depolarizing NMBs, such as Vecuronium or Rocuronium, are associated with delayed elimination and prolonged recovery time in cirrhosis, especially when repeated dosing is used [63]. On the other hand, atracurium and cisatracurium, due to their particular elimination pathways involving Hofmann degradation, require no dose adjustments and exhibit relatively uniform pharmacokinetics. Neuromuscular function monitoring is desirable in all cases [60,62]. The commonly used reversal drugs for the neuromuscular blockade, such as neostigmine/atropine, or the relatively new sugammadex, do not modify the incidence of POCD [83].

Nevertheless, for a better cognitive outcome in cirrhotic patients, the current literature recommends cautious use or even avoidance of medications such as anticholinergics, antipsychotics, benzodiazepines, or H2-receptor antagonists [60].

For better control of anesthesia depth, various non-invasive monitoring techniques might be used, that could affect the POCD rates in cirrhotic patients [1]. Recently, EEG-based (i.e., BIS^®^), multiparametric-integrated algorithm-based (i.e., NOL^®^), or regional cerebral oxygenation monitoring evaluate the amplitude of hypnosis or nociception during anesthesia, optimizing the need for anesthetics [65].

Bispectral index monitoring (BIS) is a non-invasive technique that allows intraoperative analysis of EEG signals and facilitates anesthetic titration. It is a dimensionless value, ranging from 0, which translates to an isoelectric EEG, to 100 in fully awake patients, thus evaluating the depth of anesthesia. BIS-guided anesthesia (BIS target 40–60) has been found to reduce POCD at 3 months after surgery [66,84]. The CODA trial showed that, by using BIS monitoring, 23 patients per 1000 avoided POCD occurrence [84]. Furthermore, low BIS values and prolonged deep anesthesia (BIS < 40) were found to be predictors of POCD [84]. In contrast, reduced occurrence of POCD was correlated with deep anesthesia, with a BIS target of 30–45, as opposed to light anesthesia (aiming for a BIS of 45–60), in abdominal surgery patients [85]. In patients with liver cirrhosis, a target BIS value in the range of 45–55 has proved to be beneficial in liver transplantation [86]. Considering the conflicting results from the literature with regard to the depth of anesthesia and POCD incidence, it would seem that surgery-associated tissue trauma may also have a role in POCD development [87].

The NOL index accurately characterizes nociception during general anesthesia. NOL-guided anesthesia resulted in a reduction in the total intraoperative consumption of opioids [88]. Furthermore, another study revealed that its use in the intraoperative setting allowed improvement in pain scores in the postoperative period, despite the absence of any significant difference in total opioid consumption [88,89]. Altogether, these might have an indirect role in POCD occurrence in cirrhotic patients.

Auditory evoked potentials (AEPs) are alterations in the brain activity generated by auditory stimuli. It has been proved that AEP monitoring reliably indicates the level of consciousness [90]. Thus, an RCT from 2018 concluded that AEP-guided anesthesia resulted in reduced consumption of anesthetic drugs and a lower early POCD incidence [91].

Near-infrared spectroscopy (NIRS) is a non-invasive monitoring technique that reflects blood flow changes and the oxygen balance through measurements of the regional cerebral oxygen saturation (rScO2) [92], which is closely associated with POCD development. Thus, perioperative NIRS monitoring of rScO2 has been proven to minimize the occurrence of POCD and stabilize hemodynamics [93]. Considering that both AEP and NIRS are non-invasive monitoring techniques, they could be helpful in anesthesia titration in at-risk (cirrhotic) patients.

### 3.3. Postoperative Management of Cognitive Dysfunction in the Cirrhotic Patient

Postoperative care may prove to be challenging in cirrhotic patients; maintaining cognitive status should be prioritized, even if no sufficient objective assessment tools are available for diagnosing the postoperative neurocognitive decline. Surgery and anesthesia might induce cirrhosis decompensation [60]. In addition to encephalopathy, other major complications could impact the outcome: ileus, infection, allergies to antibiotics, bleeding, coagulopathy, renal or respiratory failure, and new onset or worsening ascites [44,61,94]. Thus, cirrhotic patients, especially those in CTP-B and C class, should be managed in the ICU in the early postoperative period [61]. Assessment of bleeding risk, close monitoring of coagulation, and cautious fluid management are of marked importance. Cirrhotic patients exhibit a hyperdynamic status, with hypotension being regarded as a normal occurrence in these cases. The general volume overload associated with reduced intravascular volume in cirrhotic patients may warrant the use of invasive monitoring [60,95]. Lactic acidosis may occur not only in the setting of inadequate perfusion but also as a sign of liver decompensation [95]. Moreover, renal dysfunction following surgery in liver disease patients may be a result of either acute tubular necrosis or hepatorenal syndrome. Thus, maintaining the intravascular circulating volume is crucial in these patients [61]. Salt restriction is required as a preventive measure for acute kidney injury (AKI), HE, or ascites occurrence. Regarding coagulation, INR has been suggested to be a sensitive test in assessing hepatic synthesis and a useful marker of hepatic decompensation. Targeting normal values and overcorrection of INR through the administration of FFP in an otherwise hemostatic patient is not required [60,95].

Any above-mentioned complication could exacerbate mHE or increase the risk of anesthesia-related cognitive deterioration. Lactulose therapy in the early postoperative period may be an optimal prevention measure [61]. The role of lactulose in the prevention of HE is widely accepted. Moreover, Umapathy et al. performed the psychometric hepatic encephalopathy score (PHES) in 102 cirrhotic patients. They were divided into two groups, the first one was composed of subjects without a history of overt HE, and the second group was made up of subjects with episodes of overt HE in their medical history but resolved at the moment of the study. They were evaluated on day one, with a second visit on day 3 and the last visit between days 30 and 60. The patients in the first group showed an improvement in PHES at the evaluation on day 3 and the last one compared with PHES acquired at the first evaluation, showing preserved learning capacity. In the second group, there was no improvement in PHES between days 1 and 3, suggesting that these subjects present a loss of learning capacity. However, these patients had improved PHES in the last evaluation, probably due to the therapy with lactulose and rifaximin [96].

A retrospective study analyzing 582 cirrhotic patients after non-hepatic surgery revealed that there were some factors that increased the risk of developing postoperative HE: a prior episode of HE, ASA class, postoperative acute kidney injury (AKI), and infection after surgery [97].

For a longer, persistent decline in cognition after surgery, liver transplantation remains the definitive treatment. You et al. studied 388 transplant patients with HE of various stages: 282 had grade 1–2 and 106 grade 3–4 HE, respectively. They noticed that subjects with more severe HE at the moment of surgery (grade 3–4) had more neurocognitive sequelae after liver transplantation- probably due to the more advanced liver disease [98]. On the other hand, there are some studies that showed an improvement in neurocognitive performance after liver transplantation [99,100]. Hopp et al. stated that the cognitive impairment associated with HE is reversible after liver transplantation, and the history of HE prior to transplantation does not affect the long-term cognitive outcome [101].

ERAS protocols as part of the preventive strategies for POCD proved their efficiency in improving patients’ outcomes in various meta-analyses and randomized controlled trials. Accelerated recovery as proposed by ERAS protocols owes its benefits to the reduction in the perioperative neuroendocrine response, which could be of particular interest in cirrhotic patients [102]. Thus, strategies that promote stress-free anesthesia and surgery and achieve the best physical and psychological status of the patients should focus postoperatively on bedside counseling and rehabilitation, avoidance of abdominal drains or nasogastric tubes, an opioid-sparing approach, early enteral feeding, and early mobilization. In an attempt to optimize postoperative care and reduce POCD occurrence, thus improving patients’ outcomes, other approaches may be useful: in-person clinic visits, both physical and cognitive exercise programs, and new tools such as virtual reality [103,104].

Multimodal analgesia with limited use of opioids as part of the ERAS protocols in the postoperative period is advisable [1]. Postoperative pain has been linked to neurocognitive decline [105,106,107]. Epidural catheters, local wound infiltration, or transversus abdominis plane blocks may prove beneficial in postoperative pain relief [1]. However, the use of epidural analgesia did not show a major impact on POCD incidence. Due to potential bleeding complications, it should be used cautiously in cirrhotic patients [60,108]. NSAIDs should be avoided in liver dysfunction as a result of their potential nephrotoxic effect, increased risk of GI bleeding, and platelet dysfunction. Acetaminophen remains a relatively safe option in these patients, but close monitoring is advisable [60].

The postoperative systemic inflammatory response associated with surgery and anesthesia may be responsible for the development of POCD. Proinflammatory cytokines directly affect the CNS and may lead to neuroinflammation and neurocognitive decline [109]. New data from the literature revealed that biomarkers such as S100B and neuron-specific enolase are involved in the pathogenesis of POCD [65,110]. Moreover, the use of sevoflurane increases the level of S-100B. Thus, the S-100B protein plays an important predictive role in POCD [74]. The selective cyclooxygenase-2 inhibitor parecoxib demonstrated a reduction in S100B and Il-6 levels and has been proven to be of use in minimizing POCD [111].

Observational studies also pointed out that POCD is correlated with high postoperative concentrations of inflammatory markers such as IL-6 or CRP. The inflammatory response correlated with the increased incidence of POCD may be targeted by corticoid therapy [65,112]. One meta-analysis showed, however, that cognitive dysfunction occurrence was not altered by prophylactic dexamethasone [87]. Conversely, a prospective randomized controlled trial revealed the potential beneficial effect of preoperative administration of methylprednisolone (10 mg/kg) in the reduction of IL-6 and TNF-α levels and its role in the prevention of POCD [74].

Gabapentin 900 mg p.o administered 1–2 h before anesthesia and continued for 3 days postoperatively was depicted as a promising intervention in reducing POCD rates. However, studies revealed that it resulted only in a decrease in opioid consumption and had no impact on POCD incidence or length of hospital stay [113]. Cholinesterase inhibitors have been proven to benefit the cognitive process and the quality of daily living. Memantine was also found to have neuroprotective properties by inhibiting glutamate excessive activation and neuronal degeneration [1,114]. Psychoactive drugs are, however, of marginal use in cirrhotic patients, considering their potential interference with the preexistent disturbance in the physiologic neuro-signaling pathways (i.e., endogenous benzodiazepine accumulation).

The use of virtual reality or occupational therapy as adjunctive treatment strategies in the postoperative period enhances the functional independence of the patients, resulting in reduced pain levels and even shorter hospitalization, as stated by recent research [114,115]. However, it is still debatable if these strategies are effective in the postoperative management of cirrhotic patients and what other measures should be applied for a better neurocognitive outcome.

## 4. Conclusions

The management of cirrhotic patients’ cognitive status in the perioperative setting has proven to be a challenging task for anesthesia providers. Liver cirrhosis is associated with high mortality and morbidity rates, especially in CTP class B and C patients. In addition to POCD, other major complications, such as coagulopathy, hemorrhage, and AKI, impose careful management during the perioperative period, with appropriate anesthetic regimens and cautious monitoring. POCD is roughly defined as decreased cognitive performance in patients undergoing anesthesia. Various risk factors, including increased age, baseline cognition level, depth of anesthesia, or postoperative pain level, were described to be involved in the development of POCD. In the cirrhotic patient, POCD is described as a “more than expected” decline in cognitive function. Numerous possible strategies for preventing and treating POCD are evaluated. The judicious use of anesthetic agents and techniques, the monitoring of the depth of anesthesia, and the application of ERAS protocols may prove to be advantageous in this setting. However, specific and targeted therapies for POCD are lacking. Therefore, the implementation of preventive strategies appears to be the optimal attitude. Further studies are needed for a better understanding of POCD, especially in cirrhotic patients.

## Data Availability

Not applicable.
